# Baseline neutrophil-lymphocyte ratio (≥2.8) as a prognostic factor for patients with locally advanced rectal cancer undergoing neoadjuvant chemoradiation

**DOI:** 10.1186/s13014-014-0295-2

**Published:** 2014-12-18

**Authors:** Lijun Shen, Hui Zhang, Liping Liang, Guichao Li, Ming Fan, Yongxin Wu, Ji Zhu, Zhen Zhang

**Affiliations:** Department of Radiation Oncology, Fudan University Shanghai Cancer Center and Department of Oncology, Shanghai Medical College, Fudan University, Shanghai, 200032 China

**Keywords:** Rectal cancer, Neoadjuvant chemoradiation, Neutrophil-lymphocyte ratio

## Abstract

**Background:**

The neutrophil-lymphocyte ratio (NLR) has been proposed as an indicator of systemic inflammatory response and may predict the clinical outcome in some cancers, such as head and neck cancer and gastric cancer. However, the value of this ratio is variable in different cancers. Studies of the relationship between NLR and both survival and response to chemoradiation have been limited with respect to locally advanced rectal cancer.

**Methods and materials:**

From 2006 to 2011, 199 consecutive locally advanced rectal cancer patients who were treated with neoadjuvant chemoradiation in the Shanghai Cancer Center were enrolled and analysed retrospectively. Tumor response was evaluated by pathological findings. The baseline total white blood cell count (WBC) and the neutrophil, lymphocyte, platelet counts were recorded. The neutrophil-lymphocyte ratio (NLR) and the relationship with clinical outcomes such as overall survival (OS) and disease-free survival (DFS) was analyzed.

**Results:**

With ROC analysis, the baseline NLR value was found to significantly predict prognosis in terms of OS well in locally advanced rectal cancer patients. A multivariate analysis identified that a cut-off value of NLR ≥ 2.8 could be used as an independent factor to indicate decreased OS (HR, 2.123; 95% CI, 1.140-3.954; P = 0.018). NLR ≥ 2.8 was also associated with worse DFS in univariate analysis (HR, 1.662; 95% CI, 1.037-2.664; P = 0.035), though it was not significant in the multivariate analysis (HR, 1.363; 95% CI, 0.840-2.214; P = 0.210). There was no observed significant correlation of mean value of NLR to the response to neoadjuvant chemoradiation. The mean NLR in the ypT0-2 N0 group was 2.68 ± 1.38, and it was 2.77 ± 1.38 in the ypT3-4/N+ group, with no statistical significance (P = 0.703). The mean NLR in the TRG 0–1 group was 2.68 ± 1.42, and it was 2.82 ± 1.33 in the TRG 2–3 group with no statistical significance (P = 0.873).

**Conclusions:**

An elevated baseline NLR is a valuable and easily available prognostic factor for OS in addition to tumor response after neoadjuvant therapy. Baseline NLR could be a useful candidate factor for stratifying patients and making treatment decisions in locally advanced rectal cancer.

## Background

Neoadjuvant chemoradiation therapy (NACRT) has been established as a standard treatment for locally advanced rectal cancer (LARC). Patients who achieve complete pathological response after chemoradiation show a better survival [1,2]. Although pathological examination and conventional clinicopathological prognostic variables remain the primary assessment, researchers have attempted to quantify tumor response to neoadjuvant chemoradiation and predict prognosis using other factors. Many studies have focused on the prognostic significance of genes, proteins and inflammatory factors, but no consensus has been reached. Identifying patients with differential therapeutic responses and prognosis according to affordable and reliable markers is significant for following risk-stratified therapy in locally advanced rectal cancer.

Recently, the local and systemic inflammatory response has been reported as an important determinant of disease progression and survival in colorectal cancer [3]. The Glasgow prognostic score, which is based on elevated circulating concentrations of C-reactive protein and hypoalbuminaemia, is independently associated with poor survival [4]. Other inflammatory parameters such as interleukins, TGFβ and VEGF have also been found to be associated with outcome [5]. However, these parameters are not routinely measured in daily clinical practice. The systemic inflammatory response measured using the surrogate neutrophil-lymphocyte ratio (NLR) has been proposed as an inexpensive and widely available marker to predict cancer patient survival [6]. Several studies have demonstrated that elevated NLR was associated with inferior survival in several common cancers, including colorectal cancer [7-9], gastric cancer [10], renal cancer [11], breast cancer [12] and pancreatic cancer [13]. The causes of this inferior survival have not yet been identified, but an elevated NLR is thought to correlate with the decline of nutrition and immune function. More specifically in locally advanced rectal cancer (LARC) patients, some studies have demonstrated that an elevated lymphocyte count is associated with increased downstaging following neoadjuvant chemoradiation [14], while elevated NLR is associated with short time to local recurrence (TTLR) and worse overall survival (OS) and disease-free survival (DFS) [15]. However, with regard to the response of neoadjuvant chemoradiation and prognosis in locally advanced rectal cancer patients, there are still relatively few studies also with small number of patients have focused on this issue. In addition, it’s very important to discriminate different risk groups up front, because this may influence the treatment modality choosing for these patients. If the patients’ baseline characteristics can be stratified into different risk groups, we may avoid over-treatment for patients with good prognosis while intensify treatment for patients with poor prognosis.

Therefore, the aim of our study is to further clarify the prognostic significance of the baseline NLR in locally advanced rectal cancer with neoadjuvant chemoradiation and its relationship with radiation response.

## Methods

### Patients and evaluation

A consecutive cohort of 224 patients with locally advanced (cT3-4 and/or cN1-2) rectal cancer treated with neoadjuvant chemoradiotherapy followed by surgery at the Fudan University Shanghai Cancer Center between January 2006 and December 2011 was identified from the colorectal cancer database. All medical records were retrospectively reviewed.

Primary tumors were staged by MRI. All patients underwent conventional radiation with concurrent 5-fluorouracil-based chemotherapy. The mean radiation dose was 50 Gy (range 45–55 Gy) with daily fraction of 1.8-2.0 Gy. Radiation treatments were performed according to the institutional protocols. For the analysis in this study, the exclusion criteria included patients who did not have rectal adenocarcinoma, were diagnosed with a non–skin cancer within 5 years of the diagnosis of rectal cancer, did not complete neoadjuvant chemoradiation, were found to have metastatic disease before or at the time of surgery or had an interval from the completion of radiation to surgery greater than 16 weeks.

In this data set, 208 patients met the criteria, and 199 patients had retrievable baseline blood sampling reports within 7 days before the start of chemoradiation. The blood routine examination was detected by fluorescence flow cytometry in our center. The total white blood cell count (WBC) and the neutrophil, lymphocyte and platelet counts were recorded. The neutrophil-lymphocyte ratio (NLR) was calculated by dividing the absolute neutrophil count by the absolute lymphocyte count. The pre-treatment carcinoembryonic antigen (CEA) levels were categorised as normal (<5 ng/ml) or elevated (≥5 ng/ml). Pathologic tumor staging of the surgical specimen was performed in accordance with the 7th guidelines of the American Joint Committee on Cancer [16]. Written informed consent was obtained from the patient for the publication of this report and any accompanying images and this study was approved by the Institutional Review Board of Fudan University Shanghai Cancer Center.

The primary endpoint of the study was overall survival (OS) and disease-free survival (DFS). The secondary endpoint was neoadjuvant chemoradiation response. OS was defined as the time from the date of surgery to death from any cause. DFS was defined as the time from the date of surgery to the date of tumor relapse (local recurrence and/or distant metastases) or death. We evaluated tumor response with both ypTNM staging and TRG score. In the ypTNM staging system, good response was defined as ypT0 to 2 without lymph node metastases, and poor response was defined as ypT3 to 4 or with identified lymph node metastases. The four-point TRG system graded on a scale of 0(complete response; no viable cancer cells) to 3 (poor response; minimal or no regression, extensive residual cancer) [16]. Patients with TRG0-1 had good response while TRG2-3 had poor response.

### Statistical analysis

Receiver operating characteristic (ROC) analysis and relative area under the curve (AUC) statistics were used, and the ratio closest to the point with the maximum sensitivity and specificity was selected as the optimal cut-off value. The NLR was correlated with the clinicopathological variables using the chi-square test or Fisher’s exact test. Continuous data were expressed as the median (range) or mean ± standard deviation (SD) and compared using an independent two-sample *t*-test. Survival curves were made using the Kaplan-Meier method, and groups were compared using the log-rank test. Univariate and multivariate analyses to identify prognostic predictors were performed using Cox proportional hazard regression models. Variables with P < 0.10 on univariate analysis were entered into multivariate analyses.

Statistical analysis was performed using the SPSS statistical software package, version 18.0 (SPSS Inc., Chicago, IL, USA). A two-sided P < 0.05 was considered statistically significant.

## Results

The baseline patient characteristics and tumor pathological factors are shown in Table 1. Seventy-five percent of the patients were male, with a mean age of 55 years (range 22–76 years). Of these patients, 185 (93%) patients had curative resection, and 14 (7%) patients had palliative surgery. Clear surgical margins were achieved in 195 (98%) patients. There were 88 (44.2%) patients who were at ypT0-2 N0 after neoadjuvant chemoradiation and 38 (19.1%) achieved pathological complete response (pCR). One hundred seventeen (58.8%) patients achieved TRG0-1. For all patients, the baseline median lymphocyte count was 1.6 × 10^9^/l (range 0.6-3.8), the median neutrophil count was 3.9 × 10^9^/l (range 1.3-15.7), and the median NLR was 2.4 (range 1.0-8.9).

**Table 1:** **Patient characteristics**

**Variables**	**N (%)**	**Low NLR (%)**	**High NLR(%)**	**P-value**
**Gender**				
Female	50 (25.1)	35 (70.0)	15 (30.0)	
Male	149 (74.9)	98 (65.8)	51 (34.2)	0.583
**Age, years**				
<60	135 (67.2)	90 (66.7)	45 (33.3)	
≥60	64 (31.8)	43 (67.2)	21 (32.8)	0.942
**Tumor stage before CRT**				
II	26 (13.1)	23 (88.5)	3 (11.5)	
III	173 (86.9)	110 (63.6)	63 (36.4)	0.012
**Tumor location**				
Low	122 (61.3)	82 (67.2)	40 (32.8)	
Middle-high	77 (38.7)	51 (66.2)	26 (33.8)	0.886
**CEA, ng/ml**				
<5	110 (55.3)	77 (70.0)	33 (30.0)	
≥5	89 (44.7)	56 (62.9)	33 (37.1)	0.292
**RT dose, Gy**				
45-50	122 (61.3)	76 (62.3)	46 (37.7)	
>50	77 (38.7)	57 (74.0)	20 (26.0)	0.087
**Surgical procedure**				
APR	115 (57.8)	81 (70.4)	34 (29.6)	
LAR	70 (35.2)	45 (64.3)	25 (35.7)	
Hartmann	14 (7.0)	7 (50.0)	7 (50.0)	0.246*
**ypTNM staging**				
ypT0-2 N0	88 (44.2)	62 (70.5)	26 (29.5)	
ypT3-4 or N+	111 (55.8)	71 (64.0)	40 (36.0)	0.334
**TRG score**				
0-1	117 (58.8)	82 (70.1)	35 (29.9)	
2-3	82 (41.2)	51 (62.2)	31 (37.8)	0.245
**Adjuvant chemotherapy**				
Yes	184 (92.5)	121 (65.8)	63 (34.2)	
No	15 (7.5)	12 (80.0)	3 (20.0)	0.393*

(*Fisher’s exact test).

Of the 199 rectal cancer patients with baseline blood sample reports, 19 (9.5%) patients developed local recurrence, 49 (24.6%) patients developed distant metastasis and 43 (21.6%) patients had died by the time of last follow-up. The median follow-up for survivors was 31 months (range 1–84 months). In the ROC analysis, the AUC (area under the curve) for NLR was 0.635 (P = 0.007) for OS and 0.569 (P = 0.107) for DFS. The optimal cut-off value for NLR was 2.8 and 3.6 for OS and DFS, respectively. Because the NLR could not statistically discriminate DFS, the cut-off value of 2.8 was chosen for further analysis. An NLR ≥2.8 was defined as high NLR, and an NLR < 2.8 was defined as low NLR in our study.

Table 1 shows the difference in clinicopathological characteristics between the NLR groups. Overall, 133 (66.8%) patients had a low NLR, and 66 patients (33.2%) had a high NLR. A high NLR was only significantly associated with a higher clinical stage before treatment. None of the other clinicopathological characteristics were found to associate with the NLR.

**Figure 1:**
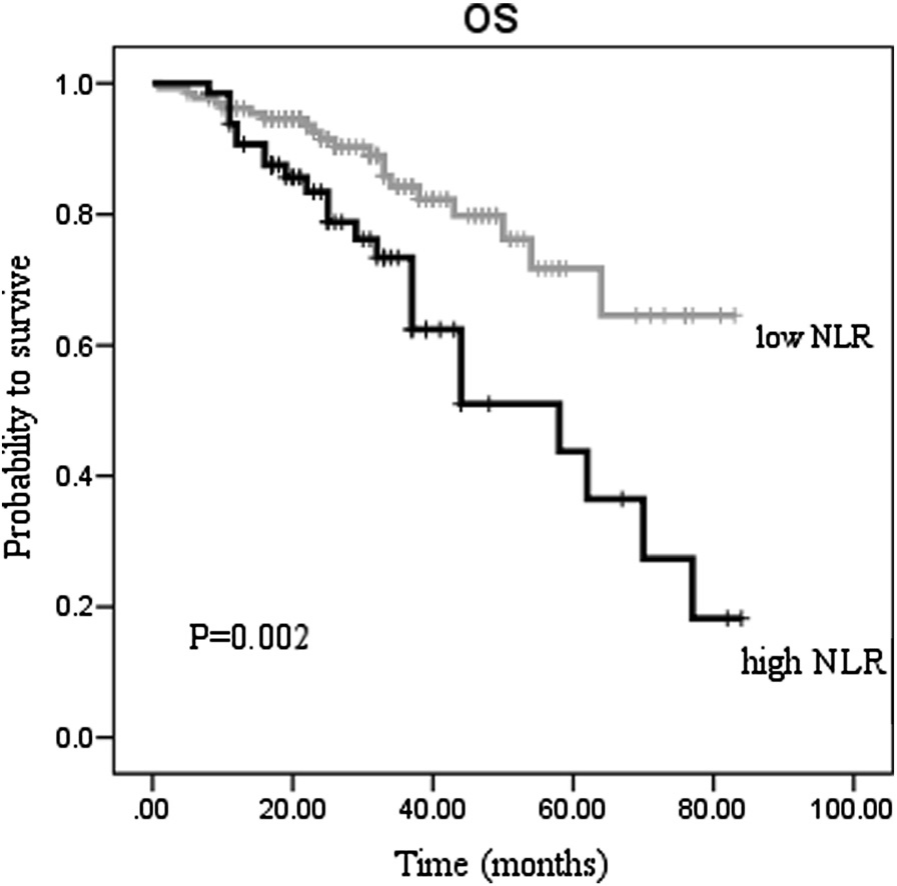
**Overall survival stratified by neotrophil-lymphocyte ratio (NLR).**

**Figure 2:**
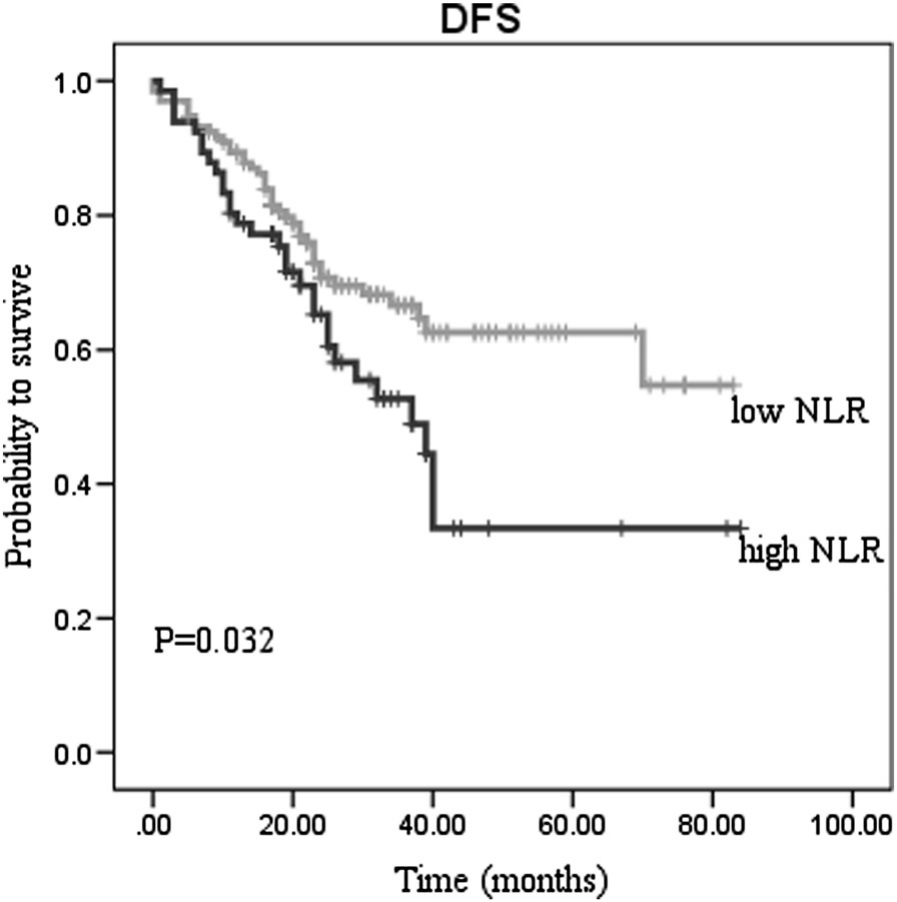
**Disease-free survival stratified by neotrophil-lymphocyte ratio (NLR).**

An elevated NLR was a significantly poor prognostic factor. The 5-year overall survival (OS) in patients with NLR <2.8 was 0.717 and 0.437 in patients with NLR ≥2.8 (P = 0.002) (Figure 1). The 5-year disease-free survival (DFS) in patients with NLR <2.8 was 0.625, and it was 0.334 in patients with NLR ≥2.8 (P = 0.032) (Figure 2).

The results of the univariate analysis are shown in Table 2. An elevated NLR before chemoradiation was significantly associated with decreased OS (HR, 2.526; 95% CI, 1.384-4.610; P = 0.003) and decreased DFS (HR, 1.662; 95% CI, 1.037-2.664; P = 0.035). A multivariate analysis was performed for the characteristics with P < 0.1 by univariate analysis (Table 3). This analysis indicated that NLR (HR, 2.123; 95% CI, 1.140-3.954; P = 0.018), ypTNM staging (HR, 3.928; 95% CI, 1.651-9.345; P = 0.002) and adjuvant chemotherapy completion (HR, 3.506; 95% CI, 1.251-9.827; P = 0.017) were associated with OS. NLR showed no association with DFS (HR, 1.363; 95% CI, 0.840-2.214; P = 0.210), and ypTNM staging (HR, 3.477; 95% CI, 1.857-6.509; P < 0.001) was the only factor associated with DFS in multivariate analysis.

**Table 2:** **Univariate analysis in relation to overall survival and disease-free survival**

**Patient characteristics**	**Overall survival**			**Disease-free survival**		
	**HR**	**95%** **CI**	**P-value**	**HR**	**95%** **CI**	**P-value**
**Age, years**						
<60						
≥60	0.954	0.488-1.865	0.891	0.729	0.431-1.234	0.240
**Gender**						
Female						
Male	1.029	0.492-2.153	0.939	0.992	0.575-1.714	0.978
**Tumor stage before CRT**						
II						
III	7.237	0.992-52.812	0.051	3.468	1.262-9.532	0.016
**Tumor location**						
Low						
Middle-high	1.321	0.720-2.426	0.368	1.025	0.635-1.656	0.920
**NLR before CRT**						
Low (<2.8)						
High (≥2.8)	2.526	1.384-4.610	0.003	1.662	1.037-2.664	0.035
**Platelets count, ×10** ^**9**^ **/l**						
<300						
≥300	0.655	0.258-1.664	0.373	0.813	0.417-1.587	0.544
**CEA, ng/ml**						
<5						
≥5	2.014	1.092-3.715	0.025	1.727	1.081-2.761	0.022
**RT dose, Gy**						
45-50						
>50	0.801	0.403-1.594	0.527	0.716	0.432-1.185	0.193
**Surgical procedure**						
Curative surgery						
Palliative surgery	2.383	0.994-5.713	0.052	2.2	1.048-4.616	0.037
**Lymphatic or vascular invasion**						
No						
Yes	2.346	1.085-5.073	0.03	2.274	1.221-4.238	0.010
**Neural invasion**						
No						
Yes	1.254	0.557-2.824	0.585	1.382	0.757-2.525	0.293
**Circumferential resection margin**						
Negtive						
Positive	1.956	0.470-8.148	0.357	1.454	0.356-5.936	0.602
**ypTNM staging**						
ypT0-2 N0						
ypT3-4/N+	4.223	1.953-9.133	<0.001	4.204	2.337-7.562	<0.001
**TRG score**						
0-1						
2-3	1.897	1.031-3.491	0.040	1.714	1.076-2.731	0.023
**Adjuvant chemotherapy**						
Yes						
No	2.354	0.982-5.639	0.055	1.294	0.592-2.826	0.518

**Table 3:** **Multivariate analysis in relation to overall survival and disease-free survival**

**Patient characteristics**	**Overall survival**			**Disease-free survival**		
	**HR**	**95%** **CI**	**P-value**	**HR**	**95%** **CI**	**P-value**
**Tumor stage before CRT**						
II						
III	3.846	0.502-29.465	0.195	2.045	0.724-5.778	0.177
**NLR before CRT**						
Low (<2.8)						
High (≥2.8)	2.123	1.140-3.954	0.018	1.363	0.840-2.214	0.210
**CEA, ng/ml**						
<5						
≥5	1.364	0.698-2.665	0.363	1.427	0.876-2.324	0.153
**Surgical procedure**						
Curative surgery						
Palliative surgery	1.289	0.488-3.401	0.608	1.472	0.680-3.185	0.327
**Lymphatic or vascular invasion**						
No						
Yes	1.419	0.618-3.257	0.409	1.368	0.712-2.629	0.347
**ypTNM staging**						
ypT0-2 N0						
ypT3-4/N+	3.928	1.651-9.345	0.002	3.477	1.857-6.509	<0.001
**TRG score**						
0-1						
2-3	0.904	0.464-1.760	0.766	1.027	0.626-1.684	0.916
**Adjuvant chemotherapy**						
Yes						
No	3.506	1.251-9.827	0.017			

We also validated the cut-off values from a previous study in our patient cohort. Carruthers et al. [15] found that an NLR ≥5 could predict outcome in patients undergoing neoadjuvant chemoradiation for locally advanced rectal cancer. In our study, a NLR ≥5 was significantly associated with worse OS (HR, 3.286; 95% CI, 1.362-7.932; P = 0.008) in the univariate analysis and remained significant in the multivariate analysis (HR, 2.849; 95% CI, 1.097-7.398; P = 0.032). For DFS, NLR ≥5 did not show statistical significance in either the univariate or multivariate analysis. However, there were only 15 (7.5%) patients with NLR ≥5 in our cohort.

The relationship of the tumor response to the mean NLR and the mean lymphocyte count was analysed. When we evaluated with ypTNM staging system, the mean NLR in the ypT0-2 N0 group was 2.68 ± 1.38, and it was 2.77 ± 1.38 in the ypT3-4/N+ group with no statistical significance (P = 0.703). The mean lymphocyte count was 1.70 ± 0.57 and 1.70 ± 0.60 for the good and poor response groups (P = 0.884), respectively. When we evaluated with TRG system, the mean NLR in the TRG 0–1 group was 2.68 ± 1.42, and it was 2.82 ± 1.33 in the TRG 2–3 group with no statistical significance (P = 0.873). The mean lymphocyte count was 1.66 ± 0.54 and 1.76 ± 0.64 for the good and poor response groups (P = 0.260), respectively. ROC analysis showed a minimal sensitivity of NLR to predict ypTNM staging (AUC = 0.526; P = 0.534) or TRG score (AUC = 0.546; P = 0.268). The chi-square test in Table 1 also showed no significant association between an NLR of 2.8 with ypTNM staging or TRG score. Therefore, NLR and lymphocyte count were unable to predict neoadjuvant chemoradiation response in these LARC patients.

## Discussion

Our study has highlighted the potential ability of NLR to serve as a prognostic marker for overall survival in locally advanced rectal cancer (LARC) patients. NLR ≥2.8 is associated with worse survival in these patients undergoing neoadjuvant chemoradiation, but no correlation was found between NLR and radiation response. Several translational studies have demonstrated that NLR can serve as a marker of systemic inflammatory and immune response and can predict clinically useful outcomes for multiple cancers, including LARC. Taken together, these results indicate that the NLR is a simple and robust laboratory variable for risk stratification in LARC patients.

The mechanisms of inflammation that promote tumor progression and immune response suppression remain under investigation. A high NLR reflects an enhanced neutrophil response and/or relative lymphopenia. Neutrophils are a type of inflammatory cell that could secrete circulating vascular endothelial growth factor (VEGF), chemokines and proteases that stimulate angiogenesis, and these inflammatory cytokines may establish a tumor microenvironment and promote tumor development and progression [17,18]. Moreover, an elevated neutrophil count may activate immunosuppression through the inhibition of natural killer cells and activated T cells [19,20]. Furthermore, lymphocytes are thought to be responsible for the adaptive immune response and participate in cancer immunosurveillance and immunoediting [21]. Tumor-infiltrating lymphocytes appear to play an anti-tumorigenic role in colorectal cancer [22]. The proportion of different subsets of T cells can also influence the clinical outcome. High densities of CD3+ T cells, CD8+ cytotoxic T cells and CD45RO+ memory T cells are typically associated with a longer disease-free survival (after surgical resection of the primary tumor) and/or overall survival [23]. Therefore, the NLR may influence the survival outcome by influencing the tumor microenvironment and immune system in several types of cancer. More specific studies are needed to combine such immunohistochemical and bio-molecular analyses.

Although there is a body of evidence suggesting that an elevated NLR indicates worse survival, the threshold of NLR ranges between 2 and 5 in different studies of colorectal cancer [24], and the relationship between NLR and tumor response to neoadjuvant chemoradiotherapy remains controversial. Carruthers et al. found that an elevated NLR was associated with worse survival, but they found no correlation between NLR and tumor downstaging [15], which is in agreement with the findings of our study. A validation cut-off value of 5, as used in their study [15], could discriminate OS in our cohort as well, but with less sensitivity. Therefore, a much larger population is needed for identifying the optimal cut-off value. Kitayama et al. analyzed 73 locally advanced rectal cancer patients retrospectively and showed that the lymphocyte ratio in WBC was significantly higher (P = 0.020) and the neutrophil ratio tended to be lower (P = 0.099) in a pathological complete response (pCR) group [14]. Krauthamer et al. reported a significantly higher probability of pCR to NACRT in patients with clinical stage III LARC patients who had an NLR value <5 (OR = 2.54; P = 0.04) by multivariate analysis, but it should be noted that only 10 patients achieved pCR [25]. Additional parameters in routine blood measurements such as platelets may have implications for the metastasis and prognosis of colorectal cancer [26]. However, we found that neither NLR nor lymphocyte counts was associated with tumor response evaluated with ypTNM staging or TRG score, and no relationship was found between platelet count and tumor response or survival. A further validation of these differences is necessary.

Morever, Kitayama J, et al. found circulating lymphocyte count was most markedly decreased in the CRT period and gradually recovered by the time of surgery, while the numbers of neutrophils were comparatively stable [14]. We have observed that the NLR was mainly influenced by concomittant chemotherapy and granulocyte colony-stimulating factors during the therapeutic process of chemoradiation. That is, the NLR would like to decrease after concomittant chemotherapy while increase after treating with granulocyte colony-stimulating factors. Therefore, the baseline NLR which is not interfered by any therapy is more important as a prognostic factor for patients.

It should be noted that although the NLR is easy to measure, its utility as a marker of systemic inflammation may be affected by many conditions, including coronary disease [27], metabolic syndrome [28], inflammatory diseases and any medication related to inflammatory conditions in the patients. These limitations must be considered because this is a retrospective study, and no specific data were available about co-morbidities and the cause of death. These variables will influence the specificity of NLR as a prognostic factor in our rectal cancer patient cohort. Another limitation is that 86.9% of the patients in our cohort with stage III have a worse 5-year OS (62%) compared with well-organised randomized clinical trial patients (76%) [29]. This result is likely because our cohort is a routine locally advanced rectal cancer population without strict inclusion criteria for neoadjuvant chemoradiation. Although there was no significant difference in the DFS between the NLR groups according to the multivariate analysis, the difference may be significant with a longer follow-up time and a larger patient population.

Even considering these limitations, our data indicate that an elevated pre-treatment NLR may serve as a prognostic marker for risk stratification in locally advanced rectal cancer patients who received neoadjuvant chemoradiation. In addition, tumor response, which is the tumor stage after treatment, and the ability to receive adjuvant chemotherapy remain the most significant prognostic factors for overall survival in the multivariate analysis. In this study, the NLR did not represent a alternate factor for either of them. Although systemic adjuvant chemotherapy is an independent prognostic factor for OS in our study, the benefit of adjuvant chemotherapy remains controversial. The EORTC 22921 study showed that the addition of adjuvant chemotherapy benefited only the group of patients who had a reduction in tumor stage to ypT0-2, while there was no benefit in the group who had no tumor reduction (ypT3-4) [30]. The ongoing NCT01941979 study is the first phase III randomized trial comparing adjuvant chemotherapy and observation for rectal adenocarcinoma patients with ypT3-4, N0-1 after neoadjuvant chemoradiotherapy. The investigator believes that this subgroup of patients will benefit from adjuvant therapy [31]. Therefore, it may make sense for patients with elevated NLR to receive intensive preoperative treatment because the postoperative chemotherapy remains controversial.

## Conclusion

In conclusion, our study demonstrated that the pre-treatment NLR may be an independent, significant predictor of long-term mortality in locally advanced rectal cancer patients undergoing neoadjuvant chemoradiation. Large-scale prospective studies and a validation study are warranted to confirm the optimal NLR cut-off value and its prognostic significance.
